# Therapeutic application of light and electromagnetic fields to reduce hyper-inflammation triggered by COVID-19

**DOI:** 10.1080/19420889.2021.1911413

**Published:** 2021-04-29

**Authors:** Marootpong Pooam, Blanche Aguida, Soria Drahy, Nathalie Jourdan, Margaret Ahmad

**Affiliations:** a Photobiology Research Group, Sorbonne Université – CNRS, Paris, France; b Department of Biology, Faculty of Science, Naresuan University, Phitsanulok, Thailand; c Xavier University, Cincinnati, Ohio, U.S.A

**Keywords:** COVID-19, photobiomodulation therapy, electromagnetic fields, reactive oxygen species, inflammation, cytokine storms

## Abstract

COVID-19 – related morbidity is associated with exaggerated inflammation and cytokine production in the lungs, leading to acute respiratory failure. The cellular mechanisms underlying these so-called ‘cytokine storms’ are regulated through the Toll-like receptor 4 (TLR4) signaling pathway and by ROS (Reactive Oxygen Species). Both light (Photobiomodulation) and magnetic fields (e.g., Pulsed Electro Magnetic Field) stimulation are noninvasive therapies known to confer anti-inflammatory effects and regulate ROS signaling pathways. Here we show that daily exposure to two 10-minute intervals of moderate intensity infra-red light significantly lowered the inflammatory response induced via the TLR4 receptor signaling pathway in human cell cultures. Anti-inflammatory effects were likewise achieved by electromagnetic field exposure of cells to daily 10-minute intervals of either Pulsed Electromagnetic Fields (PEMF), or to Low-Level static magnetic fields. Because current illumination and electromagnetic field therapies have no known side effects, and are already approved for some medical uses, we have here developed protocols for verification in clinical trials of COVID-19 infection. These treatments are affordable, simple to implement, and may help to resolve the acute respiratory distress of COVID-19 patients both in the home and in the hospital.

## 1. Introduction

COVID-19 induces Acute Respiratory Distress Syndrome in about 15% of cases. Mortality has been associated with the so-called ‘Cytokine Storms’, a hyperactive immune response leading to excess production of cytokines causing damage to lung tissues. Targeting this hyper-inflammatory response to reduce excess cytokine production is therefore proposed as an effective therapy to reduce mortality rates [1]. Nonetheless, currently known anti-inflammatory drugs have not proven clinically effective [1].

### 1.1 Photobiomodulation therapy and inflammation

The beneficial effects of red and infrared light in the treatment of inflammation have been well known since the time of Niels Finsen, who received the Nobel Prize in 1903 for his demonstration that a human autoimmune disease, *lupus vulgaris*, could be successfully treated and cured by application of visible light [2]. In subsequent years, illumination of patients with red or infrared light, known as Photobiomodulation therapy (PBM), was proven clinically effective against a variety of human diseases including Achilles tendinopathy [3], Alopecia Areata [4–6], psoriasis [7–9], thyroiditis [10, 41], and arthritis [11–13]. A unifying feature of all of these conditions is that they involve excessive inflammation [3,5,9,14]. In particular, thyroiditis and psoriasis are both autoimmune conditions caused by an exaggerated host immune response [9,10].

These clinical findings are supported by studies *in vitro* which examine the progress of inflammation in cell cultures by assaying cellular markers for inflammation such as cytokine synthesis [15]. Importantly, PBM therapy has been reported effective against acute lung inflammation (ALI) in an animal (rat) disease model analog of Acute Respiratory Distress Syndrome (ARDS) similar to that caused by COVID-19 in humans [16]. These and other observations have led to the recent suggestion that PBM therapy may serve as a potential treatment for hyper-inflammation and mortality caused by COVID-19 infection [17,18,19].

The wavelength range used for PBM therapy currently spans the infrared and red-light spectral regions. Exposure to these wavelengths is not known to generate any harmful side effects and infrared light, in particular, is the wavelength which best penetrates body tissue. Exposure devices are commercially available in a large range of intensities and wavelengths; they are fitted with laser or high-output LEDs as optical sources and exposure duration can be adjusted.

### 1.2 Pulsed electromagnetic field (PEMF) therapy and inflammation

Exposure to externally applied electromagnetic fields, known as Pulsed Electromagnetic Field (PEMF) therapy [19,20] has likewise been shown to have anti-inflammatory properties in a range of diseases [21–24]. PEMF therapy involves the external application of low intensity pulsed magnetic fields from 10 to approximately 300 Hz in frequency and around 10 mTesla maximum magnetic field strength. Unlike light, PEMF signals can penetrate the human body efficiently to reach internal organs, e.g., bronchi and lungs. Clinical studies have shown the effectiveness of PEMF in treating such conditions as arthritis [25,26], chronic pain [21,22,27,28], bone injury [29–31], wound healing [32–36], and lupus erythematosus [37]; all of these PEMF treatments involve the resolution of underlying inflammatory conditions [38,39,40]. In the United States, several PEMF devices used for bone healing have been FDA approved for decades.

A more direct analysis of PEMF therapy on the progress of inflammation has been obtained from recent molecular studies using different mammalian cell culture models *in vitro* [23,26,38,39,40,41]. PEMF exposure was found to induce marked decrease in pro-inflammatory cellular markers including cytokines, gene expression, and activation of NFkB which characterize both the acute and the chronic phases of inflammation. Some changes occurred rapidly within hours after PEMF exposure, consistent with a direct effect on the cellular immune response. Intriguingly, many of these clinical applications of PEMF (electromagnetic) therapy seem to overlap with those of PBM (illumination) therapy, in particular, those involving the resolution of inflammatory conditions.

Therefore, both PBM and PEMF exposures show potential for application to COVID-19 therapy.

### 1.3 Optimizing PBM and PEMF therapy protocols

In this study, we wished to establish whether illumination or electromagnetic field therapy may be effective specifically against COVID-19 induced inflammation. This hyper – inflammatory response, which gives rise to Severe Acute Respiratory Distress as caused by SARS COVID-19, is characterized by accumulation of inflammatory cells, edema formation, and a significant increase in inflammatory cytokines [1]. The specific immune mechanism underlying this pathology has been well characterized and shown to involve the Toll-like Receptor 4 signaling pathway. Activation of TLR4 pathway by pathogen elicitors stimulates cellular adaptors TRIF, activates NF-kB and other downstream regulators, and up-regulates synthesis of cytokines including Il-6, IL-1b, and IL-8 to create pathological ‘cytokine storms’ [42]. The important feature of the TLR-4 dependent inflammatory response is its high conservation among different cell types and even in different mammalian species. A significant effect of PBM or PEMF on this response in cell culture is therefore a good indication for potential therapeutic effectiveness in patients.

In this study, we have tested the effect of infrared light exposure, PEMF exposure, and static magnetic field exposure on a cell culture system specifically engineered to initiate an inflammatory immune response through the Toll-like receptor 4 (TLR4) signaling pathway (HEK – TLR4; see methods) responsible for the acute respiratory distress syndrome induced by lung pathogens [42]. In this cell culture system, a secreted colorimetric substrate (SEAP) is produced under the control of NF-kB and AP1, which are central regulators of the inflammatory response induced through TLR4 activation, and are required for cytokine overproduction. This system allows for rapidly and efficiently testing the effectiveness of multiple treatment conditions for their anti-inflammatory effects with relevance to clinical outcomes.

## 2. Materials and methods

### 2.1 Cell culture conditions

Human embryonic kidney HEK293 cell lines https://www.invivogen.com/hek-blue-htlr4, stably expressing human TLR4 (InvivoGen, San Diego, CA, USA), were used for all experiments. HEK-TLR4 cells express an alkaline phosphatase (AP) reporter gene regulated by NF-κB and AP1 transcription factors. The quantification of cell-infection was measured by assaying alkaline phosphatase activity in cell culture medium containing colorimetric enzyme substrates.

Cells were cultured in DMEM high glucose (Dulbecco′s Modified Eagle Medium; DMEM, Sigma, St Louis, MO) containing 4500 mg/l of glucose, 10% (v/v) heat-inactivated fetal bovine serum (Gibco, Dublin, Ireland) and 1x HEK-Blue Selection solution (InvivoGen, San Diego, CA, USA) and grown at 37°C under a humidified atmosphere at 5% CO_2_ in a dedicated incubator (MCO-18AC, Panasonic Biomedical, Leicestershire, UK).

Cells were first amplified in 75 ml culture flasks and sub-cultured every 4 days. For experimental trials, HEK cells were seeded from a single stock culture flask at a density of 10^4^ cells per well in 96-well plates. Inflammatory response was stimulated 4 hours after seeding by incubation with bacterial lipopolysaccharide (LPS) dissolved in Phosphate Buffered Saline (PBS) (Sigma, Mo. USA). A final concentration of 100 ng/ml LPS was used for all tests. Negative control cultures were obtained by adding inert physiological saline (PBS) at the same volume as the LPS substrate to the control culture medium. After LPS addition, cell cultures were incubated for a further 16 hours before transfer to the relevant exposure conditions (LLF, PEMF or Infrared light). All cultures were grown in parallel from the same cell stock culture and under identical conditions.

### 2.2 Low-level magnetic field exposure conditions

Two types of electromagnetic fields, Low-Level static magnetic field (LLF) and pulsed electromagnetic fields (PEMF) were used as treatment conditions. The LLF in this study was generated by a Mu-metal cylinder 15 cm in diameter and 30 cm in height with 1 mm of thickness placed within the incubator. The 96-well culture plate was placed at the center of the Mu-metal cylinder where the intensity of the static magnetic field was less than 2 μT as measured with a Fluxgate magnetometer Mag-03 H (Barrington Instruments, Oxford, UK). The LLF condition was applied for either 10 min every 12 h over a 48-hour interval, or else continuously for 48 h. The sham condition for LLF treatment was performed as described previously in [1] A Helmholtz coil (10 cm diameter, at a separation of 10 cm) was inserted into the μ-metal cylinder. Each coil consisted of 20 windings of copper-wire of 1 mm diameter. The current provided to the coils generated a 40 μT static MF, which is the strength of local magnetic field inside the incubator. For each exposure condition, 5 duplicate wells were analyzed and averaged to obtain the results for a single experimental measurement.

### 2.3 PEMF exposure conditions

The PEMF stimulus was generated by an E-cell device (EC10701; GEM Pty Ltd., Perth, Western Australia). The frequency, intensity, and signal shape characteristics have been fully documented in our former studies [see Supplement, 43]. The device contains a coil with horizontal dimensions of 9 × 5.5 cm and 200 turns. The top of the device was placed 12 mm below the 96-well plates for the PEMF exposure. Details of the relevant controls for temperature and possible artifactual effects are all as previously described [See supplement, 43]. During the 10-minute stimulation period, PEMF was applied at a frequency of 10 Hz and with peak magnetic intensity of 1.7 mT, around 40X higher than the Earth’s magnetic field.

In our experiments, the PEMF exposure was applied for 10-minute intervals at a time, and given repetitively every 12 h for 48 h. Control cells were grown in an identical manner (induction of inflammation by 100 ng/ml LPS) but maintained without exposure to either LLF or PEMF magnetic field stimulation. For each exposure condition, 5 wells were individually analyzed and averaged to provide a single data point in a given experiment.

For the sham PEMF experiments, the control condition (growth in the incubator under no applied magnetic field – see above) was compared to that of a sham PEMF treatment induced through a reverse-wound PEMF device. The reverse wound coil device provides the same current as the test PEMF signal, however produces no pulsed magnetic field; this control is fully documented previously [43, see supplement Fig.7]. Cell cultures grown under control conditions were compared to the sham PEMF exposed cultures.

### 2.4 Infrared exposure conditions

Infrared exposure was achieved using two different illumination methods. For the cell culture experiments, a pre-mounted 7 – LED array of Far Red (720 nm) Rebel LEDs mounted on a SinkPAD-II 40 mm Round 7-Up base with output of 780 mW@350 mA (cat. No. SP – 02– D4 LED module) purchased from Luxeon Star LEDs, Alberta, Canada. The wavelength range was between 720 and 750 nm. The LED array was placed 20 cm above the 96-well culture plate to create a uniform beam for illumination of the culture plates. Infrared light intensity was detected by a Quantum light meter (LI-185B, Li-Cor, Lincoln, NE, USA) with a pyranometer probe (Li-Cor, Lincoln, NE, USA).For experiments involving penetration of pork ribs and muscle tissue, the Cool – IR 720 nm High Output Floodlight and Cool IR 720 nm High Output bulb (https://paris.craigslist.org/fod/d/custom-led-lightbulbs/7301609363.html) was obtained from Synlyte, Massy Palaiseau, France (synlyte@synlyte.com).

Activation of the LEDs during cell culture was controlled by a custom–built automated programmable switch as described previously (Pooam et al, 2019). Briefly, the switch was created using a 4-channel 5 V power relay board using GPIO pins of a Raspberry Pi 3B Light Starterkit to power on infrared LED illumination. The infrared sequence was programmed to switch on for 10 min. every 12 h over a total time of 48 h. The control condition was performed in an identical manner (inflammation was induced with 100 ng/ml LPS), and cultured in the darkness without infrared illumination.

### 2.5 Alkaline phosphatase assay for monitoring inflammation

The inflammatory response of HEK-TLR4 cells was measured by determining the enzyme activity of the secreted alkaline phosphatase (SEAP) reporter gene, which was normalized to the total concentration of cells per well. SEAP enzyme activity was assayed at the end of the 48-hour growth period by removing 20 µl of cell culture media from each of five duplicate wells subjected to the treatment condition. The culture media samples were then mixed with 180 µl of QUANTI-Blue^TM^ detection solution (Invivogen) which contains the AP colorimetric substrate and incubated in accordance with manufacturers specifications (30 minutes at Room Temperature) in a fresh 96-well plate. Alkaline phosphatase activity was measured as the absorbance of the detection solution at 620 nm using an Epoch microplate reader (BioTek, Winooski, Vermont, USA). Values from five duplicate wells were averaged to obtain a single experimental data point.

In order to detect possible differential cell growth effects resulting from these treatments, the HEK-TLR4 cells were also measured for total protein concentration in each well after the treatment period, using the DC Protein Assay kit (Bio‐Rad Laboratories, Mississauga, ON, Canada). Briefly, the culture medium was removed from each of the 5 duplicate wells subjected to experimental conditions. 20 µl of cell lysis buffer (25 mM Tris-HCl pH 7.4, 150 mM NaCl, 1% NP-40, 1 mM EDTA, 5% glycerol) was added to the cells inside the culture wells to induce cell lysis and achieve protein solubilization. The total lysate was then transferred into a fresh 96-well plate and mixed with the DC protein assay reagents as recommended by the manufacturer. The levels of total proteins were measured by absorbance at 750 nm by an Epoch microplate reader (BioTek). The absorbance value of QUANTI-Blue^TM^ Solution (OD620), representing secreted alkaline phosphatase activity, was subsequently normalized to the total protein concentration (OD750) and presented as a ratio (OD620/OD750).

A background level of alkaline phosphatase secretion was observed in cell cultures that had not been exposed to LPS after the 48-hour incubation period, which did not respond to anti-inflammatory treatments. This background SEAP value was subtracted from the values obtained from the LPS-stimulated cell cultures to obtain the TLR-4 dependent component of the inflammatory response. The effect of treatments is expressed as the percentage of inflammation achieved after LPS induction in IR, PEMF, or LLF treated groups as compared to the SEAP secretion response of untreated control cells that had received LPS stimulation.

### 2.6 Statistical analysis

For each individual experiment, the data from 5 duplicate wells at the exposure conditions were averaged with determination of SD and SEM. Each experiment was then repeated for a total of five biological replicates, which were used for statistical analysis. Data were analyzed using GraphPad Prism version 7.4.2 for Mac (GraphPad Software, La Jolla California, USA). Results are expressed as the mean ± standard error of the mean (SEM). The percentage of inflammatory response in the control (LPS stimulated, but not exposed to treatments) samples were compared to each of the treatment group/or sham group that had undergone therapeutic intervention (LLF, PEMF, infrared or Sham PEMF and Sham LLF). Comparisons were made among cultures grown at the same time from the same original cell stock. Data were analyzed for normality with the Shapiro–Wilk test and the equality of group variances with Brown-Forsythe test. The difference between treated and control conditions for the inflammatory response was compared by using One-way ANOVA analysis and followed by Holm-Šídák’s multiple comparisons test. Differences were considered statistically significant with a p-value < 0.05 (*), < 0.01 (**).

## 3. Results

For our experiments, we have used a commercially available engineered laboratory HEK (human embryonic kidney) cell line that permits easy monitoring of the inflammation response via a simple colorimetric assay (see methods). It incorporates the TLR4, MD-2 and CD14 co-receptor genes which confer induction of the inflammatory response by addition of a bacterial lipopolysaccharide extract to the culture media. The cell line is further stably transformed with a secreted reporter gene (SEAP – secreted embryonic alkaline phosphatase) under the control of an inducible promoter activated by AP1 and NFk1, which are global regulators of inflammatory responses including the ‘cytokine storms’ caused by COVID-19. The concentration of the secreted SEAP in the culture medium can be monitored by a colorimetric substrate detected spectroscopically by absorbance at O.D. 620 nm (see methods). As a consequence, this cell line provides a robust model for the mechanism of inflammation that occurs in lung alveolar cells.

**Figure 1.**
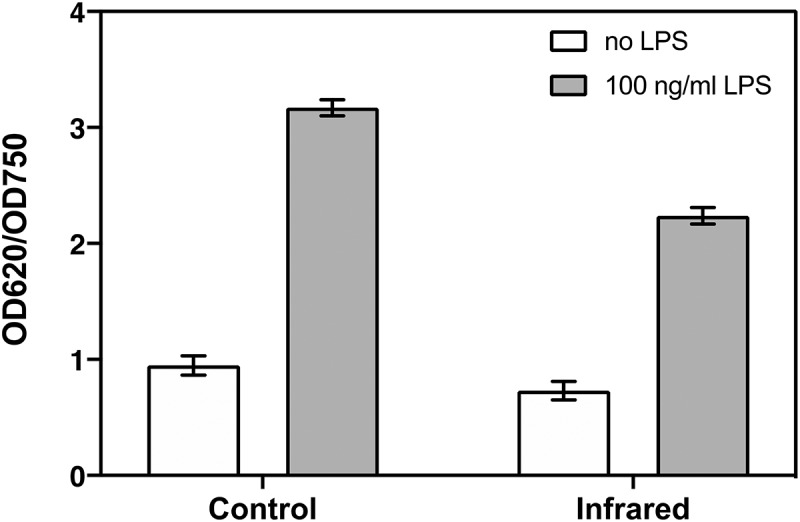
Induction of inflammation in HEK-TLR4 cells. HEK-TLR4 cell cultures were treated with or without 100 ng/ml LPS to induce inflammation as described (see Methods). The Control cell cultures (left panel) were subsequently maintained in the dark in the incubator for 48 hours without further intervention. The Infra-Red treated cells (which included both LPS induced and non-induced cultures) were exposed to 720 nm Infrared light pulses for 10 minutes at 6 W/m2, at 12-hour intervals over a two day growth period(right panel). The inflammatory response (OD620/OD750) was quantified as the absorbance of the QUANTI-Blue SEAP substrate at 620 nm. normalized to the total protein concentration(absorbance at 750 nm)(see Methods). Infrared light was successful in reducing inflammation in LPS – stimulated (Grey Bars) but not unstimulated control cells (White Bars). Data are shown as the mean ± SE taken from five individual wells of one representative biological experiment

In our initial experiments, we tested the inflammatory response of HEK cell cultures in response to 100 ng/ml lipopolysaccharide stimulation (Figure 1, left panel). The inflammatory response is measured by the secretion of SEAP, whose concentration can be monitored at the conclusion of the 48 hour experimental period using a colorimetric substrate that is added to the culture media (Methods). To avoid possible bias due to differential cell growth, SEAP expression was normalized to total cellular protein concentration from the lysed cells in each well. The results (Figure 1) showed that there was significant stimulation of SEAP levels by LPS addition, as seen by the difference compared to the basal levels in control cells (compare white to gray bar, right panel). Thus, the cell culture system provides a means to measure the inflammatory response induced via a signaling pathway implicated in onset of respiratory distress.

We next tested the effect of a single intensity of IR exposure on the inflammatory response (Figure 1, right panel). LPS– stimulated cell cultures were exposed to Infrared light generated by high-output LEDs at an intensity of 6 W/m^2^ for 10 minutes at 12-hour intervals over a period of two days (see Methods). Following IR exposure, a marked decrease in the inflammatory response was observed in LPS induced cells (gray bar, compare left to right panel). Preliminary experiments showed that optimal decrease in the inflammation response was observed when IR exposure occurred every 12 hours; once a day or more frequent exposures were less effective. However, even when provided at the optimal condition of once every 12 hours, IR exposure caused no significant change in the basal SEAP secretion from cells that had not been induced with LPS (compare white bars, left and right panels). Therefore, IR exposure reduces inflammation triggered by activation of the TLR-4 dependent signaling pathway in these cell cultures, but has no significant effect on healthy cells.

**Figure 2.**
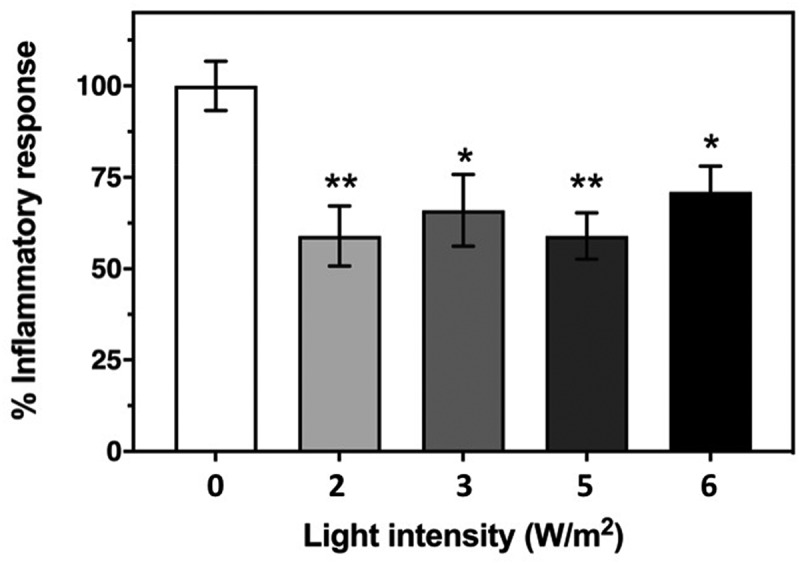
**Effect of Infrared Light exposure on the HEK-TLR4 inflammatory response**. The inflammatory response was induced in cell cultures by incubation with 100 ng/ml LPS and followed by exposure to 720 nm infrared illumination at the indicated intensities as described in Methods. The control condition represents inflammatory response of HEK-TLR4 cells exposed to 100 ng/ml LPS in the incubator with no exposure to infrared light. The decrease in the induced inflammatory response after exposure to IR light is expressed as a percentage of the Control. Data represent the mean ± SE of five independent experiments (N = 5). The asterisks indicate significance level of the differences: *p-value < 0.05 and **p-value < 0.01

An important question concerning the effectiveness of IR treatments is determining the optimum dose. Figure 2 reports the results of IR treatment at 10-minute exposures once every 12 hours, and at IR light intensities varying from 2 to 6 W/m^2^. The inflammatory response is expressed as a percentage of that measured in the untreated control (white bar). All of these intensities resulted in a 30–40% decrease in the TLR-4 induced inflammatory response. There was little change in overall effectiveness at light intensities that varied over this entire intensity range, although effects were more scattered at the lower light intensities. This suggests that raising the light intensity above the minimum required to produce an anti-inflammatory response provides neither added benefit nor harm.

We next investigated the efficacy of electromagnetic fields on the progress of inflammation in HEK – TLR4 cell cultures. We first tested the effect of Low-level static magnetic fields, since lowering the magnetic field strength significantly below the Earth’s magnetic field (40μT inside the incubator) has been previously reported to modulate cellular ROS signaling in cellular cultures [44]. A Mu-metal funnel placed inside the incubator was used to create a low-level magnetic field of less than 2 μT at its center (see Methods). Cells were placed into this funnel for LLF exposure treatments. HEK-TLR4 cell cultures were treated with LPS to induce the inflammatory response and subsequently placed in this funnel either continuously for 48 hours, or for short 10-minute exposure periods every 12 hours.

**Figure 3.**
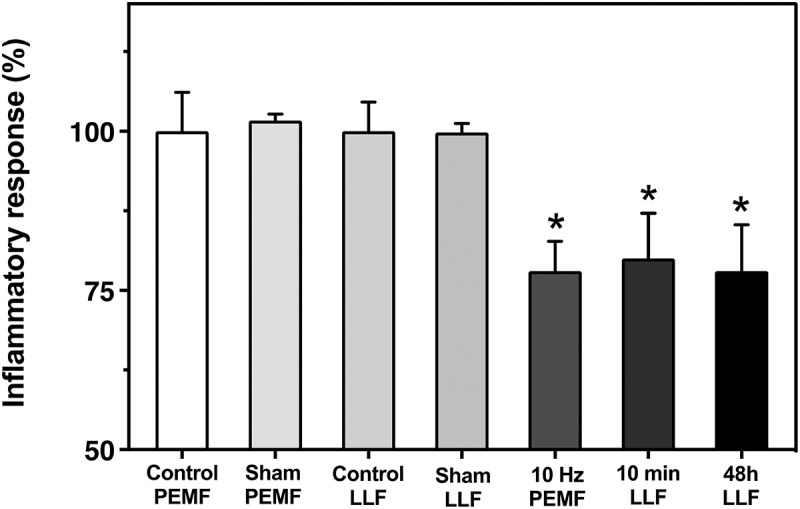
Effect of low-level static magnetic field (LLF) and pulsed magnetic field (PEMF) on HEK-TLR4 inflammatory response. The inflammatory response is shown after incubation with 100 ng/ml LPS and exposure to PEMF condition (10 Hz PMF) or LLF condition for 10 min/12h for 48h (N=4; 10 Hz LLF) or continuously for 48h (N=5; 48h LLF) in comparison to the Control (magnetic field inside the incubator of 40mT) for each condition (Control PMF, Control LLF). The Control condition represents the inflammatory response of HEK-TLR4 induced by 100 ng/ml LPS, after 48 hours growth in the incubator at the local geomagnetic field with no exposure to LLF (see methods). The sham for PEMF and LLF conditions (See methods) also present and they have no significantly different when compared to their Control conditions.  Data are shown as mean ± SE. The asterisks indicate significance level of the differences: *p-value < 0.05

The results are shown in Figure 3. When cells were exposed to a LLF for 48 hours, they consistently showed a 20% decline in the inflammatory response as compared to control cell cultures. Importantly, the same effect was found when exposure to Low-Level fields was for only 10- minute intervals, given once every 12 hours. Therefore, simply reducing of the Earth’s magnetic field for short periods can reduce the inflammatory response mediated by a signaling mechanism related to COVID-19 infection.

A widespread form of electromagnetic field stimulation has involved the application of PEMF (pulsed electromagnetic field) devices, which are currently in medical use for a variety of illnesses (see Introduction). In this study, we used a device emitting at a frequency of 10 Hz (Methods) which has been previously shown to modulate the concentration of intracellular ROS in HEK cell cultures [43].

To test a possible effect of PEMF on inflammation, we induced HEK-TLR4 cell cultures with LPS to induce the inflammatory response and subsequently exposed them to Pulsed Electromagnetic Fields for 10-minute intervals every 12 hours, over a 48-hour period (Figure 3). This treatment was found to significantly reduce (by 20%) the inflammatory response as compared to the untreated control condition. By contrast, mock-exposed control samples showed no significant difference as compared to untreated controls (Figure 3).

In sum, our results have shown that both light (Photobiomodulation therapy) and electromagnetic fields (either Static or Pulsed Electromagnetic Fields) can be effective in reducing inflammation related to the TLR4 – dependent signaling pathway in human cell cultures. Their efficacy ranges from 20% (electromagnetic fields) to 40% (Infrared) reduction in inflammatory markers over a 48-hour period.

## 4. Discussion

In this work, we provide evidence for anti-inflammatory effects of both photobiomodulation and electromagnetic fields in human cells relevant to the COVID-19 pandemic. In particular, we show that both treatments reduce the response induced through the TLR4 receptor signaling pathway, which has been directly implicated in the onset of Acute Respiratory Distress syndrome and cytokine storms associated with viral pathogens [42]. These treatments should therefore be effective against the hyper – inflammation in alveolar cells resulting from COVID-19 infection.

### 4.1 Current medical status of PEMF and PBM therapy

Both PEMF and PBM treatment are noninvasive, cost-efficient, do not require hospitalization, and have shown clinical effectiveness in reducing inflammatory conditions. Most importantly, no negative side effects have been detected and both PEMF and PBM devices have been approved for certain therapeutic purposes.

Nonetheless, they are not considered ‘mainstream’ therapies and are not widely used to treat infectious diseases. There are several reasons for this. Firstly, there are effective vaccines and pharmaceutical agents that target most pathogens, so there is no need for interventions that simply improve survival without attacking the pathogen. Secondly, underlying biochemical mechanisms have not been understood sufficiently, retarding their acceptance by the medical community. And thirdly, because of the vastly differing protocols and device types used in different studies, there has been inconsistency in the published findings as to their clinical effectiveness.

All of these difficulties have in principle been addressed in recent years. Firstly, there are few known effective, affordable drug therapies for COVID-19 at this time [1], the testing of alternative therapies seems to be particularly pertinent.

Secondly, recent studies have addressed the underlying biochemical mechanisms that mediate therapeutic benefits, by demonstrating that both light and electromagnetic field exposure treatments result in the modulation of intracellular ROS (reactive oxygen species). In PBM therapy, this can occur as a byproduct of respiration stimulated through mitochondrial cytochrome absorption of long-wavelength light [15,45], whereas magnetic fields can induce ROS through quantum physical effects on cellular redox chemistry [46, 43, 44]. Although at high concentrations, ROS are toxic, at the much lower physiological concentrations maintained in living cells, ROS are essential signaling intermediates regulating numerous cellular defense and repair pathways. This phenomenon, referred to as ‘redox biology’ [47], can now explain many of the beneficial effects of PEMF or PBM stimulation, for example, in regenerative medicine, wound healing, and resolution of both chronic and acute inflammation, all of which involve ROS signaling pathways. The role of ROS signaling in immune response is complex and can involve both positive and negative effects. However, ROS are required for bringing under control of the hyper-inflammatory response in some model systems [48,49]. As a consequence, slight changes in cellular ROS as caused by PEMF or PBM stimulation may be the means whereby they could play a beneficial role in down-regulating the hyper-inflammatory response and perhaps other intermediate steps.

Thirdly, technological advances regarding new sensors and markers combined with digitalization have led to a much accelerated and more reliable generation of Real World clinical data. With such technologies and methods becoming readily available – some even provided as wearable devices – the efficacy of therapeutic interventions are being demonstrated faster and with superior quality and reliability. As a consequence, dose and exposure conditions (such as timing) can be adapted in a real-time mode, to each patient throughout the course of the disease to achieve optimal outcome. These tools are in the process of paving the way for personalized medicine in future therapeutic applications.

### 4.2 Infrared light exposure conditions for application to COVID-19 clinical trials

Optimal anti-inflammatory effects were obtained by 10 minutes of Infra Red exposure provided at an intensity of 6 W/m^2^ to cell cultures twice daily. This treatment achieved a reduction of 35% in the TLR4– mediated inflammatory response of exposed cells as compared to the control, untreated cell cultures over a 48-hour period (Figure 2). In our study, we used LEDs at 720 nm peak wavelength (see methods) because this wavelength shows optimal penetration of water without generating heat, unlike red light (which barely penetrates the skin) or the far-infrared wavelengths which penetrate poorly and furthermore generate enormous heat. In fact, the wavelengths currently used in PMB therapy vary greatly in different studies, and span both the red and infrared spectral regions (600–900 nm) [15]. Assuming all such treatments activate a similar cellular mechanism, wavelengths outside of the range used in this study (720 nm) may show therapeutic effects. However, exposure conditions may need to be first carefully calibrated in cell culture conditions, and even more importantly evaluated for their efficiency in penetrating deep into the chest cavity (see below).

In fact, a serious problem pertaining to the clinical effectiveness of Photobiomodulation therapy is that light may not efficiently penetrate the chest cavity. To address this question, we have experimented with pork ribs, which are similar in size to a human rib cage. For our experiments, we used commercially available high output 720 nm LED floodlights and/or 720 nm high output LED bulbs (see methods). Using either of these illumination sources, we observed that adjusting the 720 nm light intensity at the pork skin surface to 850 W/m^2^ resulted in 6 W/m^2^ penetrating the pork rib cage as well as a further 2 cm of muscle tissue placed underneath the ribs. Thus, adjusting exposure intensity at the skin surface to 850–1000 W/m^2^ range using any 720 nm light should provide a therapeutic dose to treat inflammation in lung tissue lying directly underneath. This level of intensity is in agreement with that of a laser used in a previously published Case Report of treating a COVID-19 patient with light [50]. However, in that study, the wavelength was at 810 nm.

However, the beam width for most lasers in medical use is much too narrow to cover the whole lung surface, necessitating extended exposure times which may not have been optimal in the prior study [54]. Furthermore, it is unfortunate that most commercially available LED-based photobiomodulation devices do not emit at an appropriate wavelength and/or high enough intensity range for chest therapeutic uses. Those that do achieve high intensity, such as in the case of incandescent infrared bulbs, unfortunately generate such high amounts of heat that they cannot be applied close to the skin surface. In contrast, the high-output 720 nm LED lamps used in this study provide Infrared light of the appropriate spectrum, intensity, and with a suitable broad beam, and are currently available for horticultural applications.

**Figure 4.**
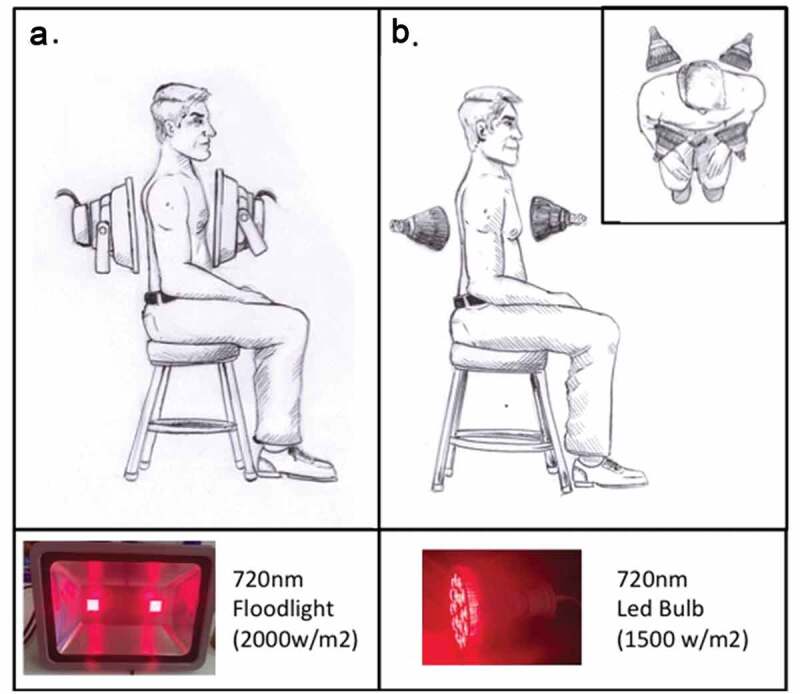
**Model for the possible application of Infrared Light in the treatment of lung inflammation**. LED light sources of the necessary 720 nm wavelength and light output intensity (achieving an intensity of 1000 W/m^2^ at the skin surface) can be applied either in the form of **A**. Two Floodlights (left panel) or as **B**. Four High Output LED Bulbs (right panel) (see Methods for specifications and origin of light sources). To provide the required 10 minute light pulses to the inflamed lung tissues at the necessary light intensity, these lamps should be positioned on both sides of the chest cavity and illuminated simultaneously to achieve maximum light penetration. The distance from the lamps to the skin surface should not exceed 5-10 cm to achieve the necessary intensity of at least 1000 W/m^2^ at the skin surface. Temperature at the skin surface using the two 720 nm Infrared Light sources does not increase beyond 5° above the ambient temperature during 10 minutes continuous illumination

A final caveat is that IR light does not efficiently penetrate bone, even at very high intensities, creating potential shadows over lung regions directly beneath the ribcage. Therefore, IR treatment should likely be the most optimum if illuminating from both sides of the patient (front and back) simultaneously, to reach maximum lung surface tissue. A suggested use of IR in the treatment of lung inflammation, using suitably positioned LED lights of the proper wavelength and intensity, is provided in Figure 4.

### 4.3 Therapeutic electromagnetic field exposure conditions for application to COVID-19 clinical trials

Problems of signal penetration do not apply to electromagnetic field treatments, which pass through the body without impediment. Both LLF exposure and exposure to a commercially available PEMF (Pulsed Electromagnetic Field) device (Methods) have proved beneficial in reducing the inflammatory response by around 20%, over a two-day period (Figures 3, 4). Electromagnetic fields seem to be somewhat less effective than IR exposure (Figure 2), and a combination of both treatments (PEMF and PBM) provided simultaneously did not result in added anti-inflammatory effects in cell cultures (not shown). However, given the more efficient penetration of electromagnetic fields through the body, a combination of PEMF and PMB therapy may prove the optimal therapeutic treatment.

A cautionary note in the use of PEMF therapy is that commercially available PEMF devices vary greatly regarding intensity, frequency, and signal shape, and do not all induce identical biological responses [51,52]. Therefore, only devices that have been properly tested and calibrated for reducing inflammation in cell cultures should be considered for clinical trials.

By contrast, Low-Level Fields are simple to produce and apply, as all that is required is a Mu metal funnel of sufficient diameter and thickness (which the supplier can recommend). A Mu-metal funnel into which a hospital bed can be inserted for 10-minute exposure intervals once every 12 hours, should be effective, and could be used in combination with Photobiomodulation to realize maximum benefit of both therapeutic strategies.

### 4.4 Combinatorial effects with anti-inflammatory drugs

An additional suggestion for therapeutic applications follows from the fact that both PBM and electromagnetic fields transiently stimulate ROS (reactive oxygen species), which are known to modulate the immune response in complex ways [53]. Drug/pharmaceutical agents cannot be applied locally and/or transiently switched on and off as in PEMF and PBM stimulation. Therefore, the cellular pathways regulated by PEMF and PMB stimulation are likely to occur by mechanisms that are complementary to those which are targeted by anti-inflammatory or anti-oxidant drugs. A promising therapeutic pathway to augment the efficacy of PBM and PEMF therapy might therefore be to combine their use with anti-inflammatory pharmacological agents.

### 4.5 Conclusion

Our experiments with InfraRed irradiation and Electromagnetic field exposure have resulted in significant reduction of inflammation in human cell cultures relating to COVID-19 induced pathology. The necessary instruments, particularly in the case of infrared light, are readily available at low cost with no toxic side effects. However, all of our work has been performed in cell cultures and, though highly suggestive, cannot represent proof of effectiveness. In fact, the only credible way to prove that this method is workable, safe, and effective is to perform carefully controlled clinical trial on COVID-19 patients who have developed lung inflammation. Given the urgency of the epidemic and its increasing cost in lives, we nonetheless hope that these initial studies will provide solid guidance to quickly translate our findings into clinical efficacy and would welcome the chance to collaborate on such future trials.

## References

[1] HHammad M, Albaqami M, Pooam M , Kernevez E , Witczak J , Ritz T , Martino C , Ahmad M . Cryptochrome mediated magnetic sensitivity in Arabidopsis occurs independently of light-induced electron transfer to the flavin. Photochem Photobiol Sci. 2020 Mar 1;19(3):341-352. 10.1039/c9pp00469f 32065192

[2] Ragab D, Eldin HS, Taeimah M, et al. COVID 19 cytokine storm; what we know So Far. Front Immunol. 2020;11:1446. 32612617 10.3389/fimmu.2020.01446 PMC7308649

[3] Roelandts R. A new light on Niels Finsen, a century after his Nobel Prize. Photodermatol Photoimmunol Photomed. 2005;21:115–117. 15888126 10.1111/j.1600-0781.2005.00160.x

[4] Bjordal JM, Lopes-Martins RA, Iversen VV. A randomised, placebo controlled trial of low level laser therapy for activated Achilles tendinitis with microdialysis measurement of peritendinous prostaglandin E2 concentrations. Br J Sports Med. 2006;40:76–80. 16371497 10.1136/bjsm.2005.020842 PMC2491942

[5] Avci P, Gupta GK, Clark J, et al. Low-level laser (light) therapy (LLLT) for treatment of hair loss. Lasers Surg Med. 2014;46:144–151. 23970445 10.1002/lsm.22170 PMC3944668

[6] Gregoriou S, Papafragkaki D, Kontochristopoulos G, et al. Cytokines and other mediators in alopecia areata. Mediators Inflammation. 2010;2010:928030. 10.1155/2010/928030 PMC2837895 20300578

[7] Gupta AK, Foley KA. A critical assessment of the evidence for low-level laser therapy in the treatment of hair loss. Dermatol Surg. 2017;43(2):188–197. 27618394 10.1097/DSS.0000000000000904

[8] Ablon G. Combination 830-nm and 633-nm light-emitting diode phototherapy shows promise in the treatment of recalcitrant psoriasis: preliminary findings. Photomed Laser Surg. 2010;28:141–146. 19764893 10.1089/pho.2009.2484

[9] Choi M, Na SY, Cho S, et al. Low level light could work on skin inflammatory disease: a case report on refractory acrodermatitis continua. J Korean Med Sci. 2011;26:454–456. 21394319 10.3346/jkms.2011.26.3.454 PMC3051098

[10] Johnston A, Xing X, Wolterink L, et al. IL-1 and IL-36 are dominant cytokines in generalized pustular psoriasis. J Allergy Clin Immunol. 2017;140(1):109–120. 28043870 10.1016/j.jaci.2016.08.056 PMC5494022

[11] Höfling DB, Chavantes MC, Juliano AG, et al. Assessment of the effects of low-level laser therapy on the thyroid vascularization of patients with autoimmune hypothyroidism by color Doppler ultrasound. ISRN Endocrinol. 2012;2012:126720. 23316383 10.5402/2012/126720 PMC3534372

[12] Brosseau L, Robinson V, Wells G, et al. Low level laser therapy (Classes I, II and III) for treating rheumatoid arthritis. Cochrane Database Syst Rev. 2005;4(2005):CD002049. 10.1002/14651858.CD002049.pub2 PMC8406947 16235295

[13] Hamblin MR. Can osteoarthritis be treated with light? Arthritis. Res Ther. 2013;15:120. 10.1186/ar4354 PMC3978432 24286607

[14] Ip D. Does addition of low-level laser therapy (LLLT) in conservative care of knee arthritis successfully postpone the need for joint replacement? Lasers Med Sci. 2015;30:2335–2339. 26420240 10.1007/s10103-015-1814-6

[15] Höfling DB, Chavantes MC, Juliano AG, et al. Low-level laser in the treatment of patients with hypothyroidism induced by chronic autoimmune thyroiditis: a randomized, placebo-controlled clinical trial. Lasers Med Sci. 2013;28:743–753. 22718472 10.1007/s10103-012-1129-9

[16] Hamblin MR. Mechanisms and applications of the anti-inflammatory effects of photobiomodulation. AIMS Biophys. 2017;4:337–361. 28748217 10.3934/biophy.2017.3.337 PMC5523874

[17] De Lima FM, Vitoretti L, Coelho F, et al. Suppressive effect of low-level laser therapy on tracheal hyperresponsiveness and lung inflammation in rat subjected to intestinal ischemia and reperfusion. Lasers Med Sci. 2013;28:551–564. 22562449 10.1007/s10103-012-1088-1

[18] Enwemeka CS, Bumah VV, Masson-Meyers DS. Light as a potential treatment for pandemic coronavirus infections: a perspective. J Photobiol Biochem B. 2020. DOI:10.1016/j.jphotobiol.2020.111891 PMC7194064 32388486

[19] Fekrazad R. Photobiomodulation and antiviral photodynamic therapy as a possible novel approach in COVID-19 management. Photobiomodul Photomed Laser Surg. 2020;38:255–257. 32326830 10.1089/photob.2020.4868

[20] Liu T, Zhang L, Joo D, et al. NF-κB signaling in inflammation. Signal Transduct Target Ther. 2017;2:17023. 29158945 10.1038/sigtrans.2017.23 PMC5661633

[21] Markov M. XXIst century magnetotherapy. Electromagn Biol Med. 2015;34:190–196. 26444192 10.3109/15368378.2015.1077338

[22] Markov MS, Nindl G, Hazlewood C, et al. Interactions between electromagnetic fields and immune system: possible mechanisms for pain control. In: Ayrapetyan S, Markov MS, editors. Bioelectromagnetics: current Concepts. Dordrecht, The Netherlands: Springer; 2006. p. 213–226.

[23] Pawluk W. Magnetic fields for pain control. In: Markov M, editor. Electromagnetic fields in biology and medicine. Boca Raton, FL: CRC Press; 2015. p. 271–297.

[24] Ross CL, Harrison BS. Effect of pulsed electromagnetic field on inflammatory pathway markers in RAW 264.7 murine macrophages. J Inflamm Res. 2013;6:45–51. 23576877 10.2147/JIR.S40269 PMC3617815

[25] Selvam R, Ganesan K, Narayana Raju KV, et al. Low frequency and low intensity pulsed electromagnetic field exerts its anti-inflammatory effect through restoration of plasma membrane calcium ATPase activity. Life Sci. 2007;80:2403–2410. 17537462 10.1016/j.lfs.2007.03.019

[26] Ganesan K, Gengadharan AC, Balachandran C, et al. Low frequency pulsed electromagnetic field–a viable alternative therapy for arthritis. Indian J Exp Biol. 2009;47:939–948. 20329696

[27] Ross CL, Zhou Y, McCall CE, et al. The use of pulsed electromagnetic field to modulate inflammation and improve tissue regeneration: a review. Bioelectricity. 2019;1:247–259. 10.1089/bioe.2019.0026 PMC8370292 34471827

[28] Guo L, Kubat NJ, Isenberg RA. Pulsed radio frequency energy (PRFE) use in human medical applications. Electromagn Biol Med. 2011;30:21–45. 21554100 10.3109/15368378.2011.566775

[29] Varcaccio-Garofalo G, Carriero C, Loizzo MR, et al. Analgesic properties of electromagnetic field therapy in patients with chronic pelvic pain. Clin Exp Obstet Gynecol. 1995;22:350–354. 8777794

[30] Bassett CAL. Therapeutic uses of electric and magnetic fields in orthopedics. In: Karpenter D, Ayrapetyan S, editors. Biological effects of electric and magnetic fields. San Diego: Academic Press; 1994. p. 13–18.

[31] Cook JJ, Summers NJ, Cook EA. Healing in the new millennium: bone stimulators: an overview of where we’ve been and where we may be heading. Clin Podiatr Med Surg. 2015;32:45–59. 25440417 10.1016/j.cpm.2014.09.003

[32] Yuan J, Xin F, Jiang W. underlying signaling pathways and therapeutic applications of pulsed electromagnetic fields in bone repair. cell physiol biochem. 2018;46:1581–1594. 29694967 10.1159/000489206

[33] Bloise N, Petecchia L, Ceccarelli G, et al. The effect of pulsed electromagnetic field exposure on osteoinduction of human mesenchymal stem cells cultured on nano-TiO2 surfaces. PLoS One. 2018;13(6):e0199046. 29902240 10.1371/journal.pone.0199046 PMC6002089

[34] Ceccarelli G, Bloise N, Mantelli M, et al. A comparative analysis of the in vitro effects of pulsed electromagnetic field treatment on osteogenic differentiation of two different mesenchymal cell lineages. Biores Open Access. 2013;2:283–294. 23914335 10.1089/biores.2013.0016 PMC3731679

[35] Maziarz A, Kocan B, Bester M, et al. How electromagnetic fields can influence adult stem cells: positive and negative impacts. Stem Cell Res Ther. 2016;7(1):54. 27086866 10.1186/s13287-016-0312-5 PMC4834823

[36] Saliev T, Mustapova Z, Kulsharova G, et al. Therapeutic potential of electromagnetic fields for tissue engineering and wound healing. Cell Prolif. 2014;47:485–493. 25319486 10.1111/cpr.12142 PMC6496472

[37] Strauch B, Herman C, Dabb R, et al. Evidence-based use of pulsed electromagnetic field therapy in clinical plastic surgery. Aesthet Surg J. 2009;29(2):135–143. 19371845 10.1016/j.asj.2009.02.001

[38] Khamaganova IV, Iu. V, Berlin VE, et al. Arutiunova, The use of a pulsed magnetic field in the treatment of lupus erythematosus. Ter Arkh. 1995;67:84–87. 8779120

[39] Kubat NJ, Moffett J, Fray LM. Effect of pulsed electromagnetic field treatment on programmed resolution of inflammation pathway markers in human cells in culture. J Inflamm Res. 2015;8:59–69. 25759595 10.2147/JIR.S78631 PMC4346366

[40] Ross C, Harrison B. The use of magnetic field for the reduction of inflammation: a review of the history and therapeutic results. Altern Ther Health Med. 2013;19(2):47–54. 23594452

[41] Ross CL, Ang DC, Almeida-Porada G. Targeting mesenchymal stromal cells/pericytes (MSCs) with pulsed electromagnetic field (PEMF) has the potential to treat rheumatoid arthritis. Front Immunol. 2019;10:266. 30886614 10.3389/fimmu.2019.00266 PMC6409305

[42] Höfling DB, Chavantes MC, Acencio MM, et al. Effects of low-level laser therapy on the serum TGF-β1 concentrations in individuals with autoimmune thyroiditis. Photomed Laser Surg. 2014;32:444–449. 25101534 10.1089/pho.2014.3716

[43] Imai Y, Kuba K, Neely GG, et al. Identification of oxidative stress and toll-like receptor 4 signaling as a key pathway of acute lung injury. Cell. 2008;133:235–249. 18423196 10.1016/j.cell.2008.02.043 PMC7112336

[44] Sherrard RM, Morellini N, Jourdan N, et al. Low-intensity electromagnetic fields induce human cryptochrome to modulate intracellular reactive oxygen species. PLoS Biol. 2018;16:e2006229. 30278045 10.1371/journal.pbio.2006229 PMC6168118

[45] Martino CF, Perea H, Hopfner U, et al. Effects of weak static magnetic fields on endothelial cells. Bioelectromagnetics. 2010;31(4):296–301. 20119972 10.1002/bem.20565

[46] Rajendran NK, George BP, Chandran R, et al. The influence of light on reactive oxygen species and NF-кB in disease progression. Antioxidants (Basel). 2019;8:640. 10.3390/antiox8120640 PMC6943569 31842333

[47] Ehnert S, Fentz AK, Schreiner A, et al. Extremely low frequency pulsed electromagnetic fields cause antioxidative defense mechanisms in human osteoblasts via induction of •O_2_^−^ and H_2_O_2_, Sc. reports. 2017;7:14544. 10.1038/s41598-017-14983-9 PMC5673962 29109418

[48] Schieber M, Chandel NS. ROS function in redox signaling and oxidative stress. Curr Biol. 2014;24:R453–62. 24845678 10.1016/j.cub.2014.03.034 PMC4055301

[49] Mittal M, Siddiqui MR, Tran K, et al. Reactive oxygen species in inflammation and tissue injury. Antioxid Redox Signal. 2014;20:1126–1167. 23991888 10.1089/ars.2012.5149 PMC3929010

[50] West AP, Brodsky IE, Rahner C, et al. TLR signalling augments macrophage bactericidal activity through mitochondrial ROS. Nature. 2011;472(7344):476–480. 21525932 10.1038/nature09973 PMC3460538

[51] Sigman SA, Mokhmeli S, Monici M, et al. A 57-year-old African American man with severe COVID-19 pneumonia who responded to supportive photobiomodulation therapy (PBMT): first use of PBMT in COVID-19. Am J Case Rep. 2020;21:e926779. 32865522 10.12659/AJCR.926779 PMC7449510

[52] Bassett CAL. Fundamental and practical aspects of therapeutical uses of pulse electromagnetic fields (PEMFs). Crit Rev Biomed Eng. 1989;17(5):451–529. 2686932

[53] Liboff AR. Signal shapes in electromagnetic therapy. In: Rosch PJ, Markov M, editors. Bioelectromagnetic Medicine. NY: Marcel Dekker; 2004. p. 17–37.

[54] Mullena L, Mengozzia M, Hanschmannb EM, et al. How the redox state regulates immunity. Free Radical Biology and Medicine 157;September 2020:3-14. 10.1016/j.freeradbiomed.2019.12.022 31899344

